# Effects of Pretreatment Processes on Grain Size and Wear Resistance of Laser-Induction Hybrid Phase Transformation Hardened Layer of 42CrMo Steel

**DOI:** 10.3390/ma18122695

**Published:** 2025-06-07

**Authors:** Qunli Zhang, Peng Shen, Zhijun Chen, Guolong Wu, Zhuguo Li, Wenjian Wang, Jianhua Yao

**Affiliations:** 1 College of Mechanical Engineering, Zhejiang University of Technology, Hangzhou 310014, China; zql@zjut.edu.cn (Q.Z.); 221122250183@zjut.edu.cn (P.S.); roll@zjut.edu.cn (Z.C.); glwu@zjut.edu.cn (G.W.); 2 Institute of Laser Advanced Manufacturing, Zhejiang University of Technology, Hangzhou 310014, China; 3 Zhejiang Provincial Innovation Center for Laser Intelligent Equipment Technology, Wenzhou 325000, China; 4 College of Materials Science and Engineering, Shanghai Jiao Tong University, Shanghai 200240, China; lizg@sjtu.edu.cn; 5 College of Mechanical Engineering, Southwest Jiaotong University, Chengdu 611756, China; wwj527@swjtu.edu.cn

**Keywords:** 42CrMo steel, laser-induction hybrid phase transformation, pretreatment process, austenite grain, wear-resistant properties

## Abstract

To address the issue of surface grain coarsening in laser-induction hybrid phase transformation of 42CrMo steel, this study investigated the effects of four pretreatment processes (quenching–tempering (QT), laser-induction quenching (LIQ), laser-induction normalizing (LIN), and laser-induction annealing (LIA)) on the austenite grain size and wear resistance after laser-induction hybrid phase transformation. The results showed that QT resulted in a tempered sorbite structure, resulting in coarse austenite grains (139.8 μm) due to sparse nucleation sites. LIQ generated lath martensite, and its high dislocation density and large-angle grain boundaries led to even larger grains (145.5 μm). In contrast, LIN and LIA formed bainite and granular pearlite, respectively, which refined austenite grains (78.8 μm and 75.5 μm) through dense nucleation and grain boundary pinning. After laser-induction hybrid phase transformation, all specimens achieved hardened layer depths exceeding 6.9 mm. When the pretreatment was LIN or LIA, the specimens after laser-induction hybrid phase transformation exhibited surface microhardness values of 760.3 HV0.3 and 765.2 HV0.3, respectively, which were 12 to 15% higher than those of the QT- and LIQ-pretreated specimens, primarily due to fine-grain strengthening. The friction coefficient decreased from 0.52 in specimens pretreated by QT and LIQ to 0.45 in those pretreated by LIN and LIA, representing a reduction of approximately 20%. The results confirm that regulating the initial microstructure via pretreatment effectively inhibits austenite grain coarsening, thereby enhancing the microhardness and wear resistance after transformation.

## 1. Introduction

The International Energy Agency (IEA) has pointed out that wind energy accounts for 36% of the growth of renewable energy, and the new installed capacity of offshore wind power is expected to reach 40 GW in 2030 [1]. Wind turbine spindle bearings are prone to fatigue pitting due to high loads, and their life span is directly related to the bearing load and material wear resistance. 42CrMo steel is widely used in the manufacturing of spindle bearings due to its high hardenability, low deformation, and excellent comprehensive mechanical properties [2]. Still, it is necessary to enhance the performance through surface quenching, and high-power bearings require the depth of the hardened layer to be more than 5 mm [3]. The current mainstream process is electromagnetic induction quenching through eddy current heating, which forms high hardness martensite but is limited by the skin effect; the depth of the hardened layer is strongly correlated with the geometry, and the alternating electromagnetic field is prone to causing edge effects [4,5,6]. Laser quenching utilizes a high-energy laser beam to achieve ultra-fast heating and cooling, forming ultra-fine-grained martensite with a narrow heat-affected zone and low thermal deformation. However, the hardened layer depth achieved by single laser quenching is typically limited to less than 2.0 mm [7,8,9], failing to meet deep hardening demands. The two processes need to be optimized to balance the hardened layer depth and thermal deformation in the strengthening of bearings.

To overcome the limitations of single heat sources, researchers in recent years have developed laser-induction hybrid heat sources by combining laser and electromagnetic induction thermal energy. Compared with single heat sources, hybrid heat sources (e.g., laser-induction hybrid cladding and laser-induction hybrid welding) exhibit significant advantages in surface modification effects [10,11,12]. However, studies on laser-induction hybrid heat sources in the field of solid-state phase transformation in metals remain relatively limited. To address the challenge of shallow hardened layers after single laser quenching, our team has developed a laser-induction hybrid quenching process [13,14,15]. This method is a hybrid quenching technique that combines laser quenching as the primary process with electromagnetic induction heating as an auxiliary source. It effectively increases the hardened layer depth while controlling thermal deformation. The authors’ previous studies have shown that the depth of the hardened layer of 42CrMo steel achieved by this hybrid technology can reach 6.8 mm [3], which effectively overcomes the defects of the large heat-affected zone and pronounced deformation of single induction quenching and meets the demand for deep hardening of high-power wind turbine bearings. However, the high heat input caused by the coupling of dual heat sources leads to significant temperature gradients, resulting in the coarsening of the surface grain (average size > 150 μm), which restricts the improvement of surface wear resistance and overall mechanical properties. Therefore, grain refinement has become a key issue to be addressed in laser-induction hybrid phase transformation.

At present, extensive research has been conducted on the refinement of austenite grains. Grain refinement is an effective means to improve the mechanical properties of materials, and the study of austenite grain growth behavior has a key role in regulating austenite grain size [16,17,18,19,20]. Austenite grain refinement during non-solidification processes can be generally classified into the following approaches: refinement through plastic deformation [21], refinement assisted by external fields (such as ultrasonic rolling and magnetic fields) [22,23], and refinement based on the inheritance effect of the initial microstructure (such as cyclic quenching) [24]. In consideration of the complexity and economic cost in practical applications, this study adopted the approach of controlling the initial microstructure to refine the austenite grain size. Wang et al. [25] used a cyclic oil quenching process on plain medium carbon steel (45 steel) to produce fine grains, and after several cycles of quenching, the grain size was reduced from 11.3 μm to 2.5 μm, and its tensile strength exceeded 1690 MPa. Huang et al. [26] found that normalizing the 28MnB5 steel phase before quenching–tempering resulted in workpieces with finer martensitic slats, and the average width of the martensitic slats was reduced from 1.5 μm to 0.9 μm, which indirectly improved the toughness. Li et al. [27] investigated the role of carbide dissolution on austenite grain growth in high-carbon, low-alloy steels and showed that carbide dissolution promoted the nucleation of both pearlite/carbide–austenite types of grains and that the growth of primary austenite grains was significantly limited during the isothermal process; the average grain size increased marginally from 65.1 μm to 69.8 μm within the temperature range of 1050–1150 °C. Yuan et al. [28] found that equiaxially reversed austenite was K-S oriented to the adjacent prior austenite grains and effectively refined the prior austenite grains from which it grew. By pre-annealing and quenching, the size of the previous austenite grains was refined to 12 µm compared to 30 µm for conventional phase transformation. Wang et al. [29] investigated the effect of different initial microstructures on the austenite grain size of alloy steels containing niobium trace elements and found that the austenite grains grown by nucleation of bainite relative to martensite and pearlite were the smallest. The above studies demonstrate that the initial microstructures play an important role in the austenite phase transformation. Nevertheless, existing research has predominantly focused on individual pretreatment methods, with limited investigation into their applicability in laser-induction hybrid quenching systems.

This study systematically investigated the effects of four pretreatment processes: quenching and tempering (QT), laser-induction hybrid quenching (LIQ), laser-induction hybrid normalizing (LIN), and laser-induction hybrid annealing (LIA) on austenite grain size and wear resistance to address the grain coarsening issue in laser-induction hybrid quenching of 42CrMo steel. By comparing the nucleation density, grain boundary distribution, and dislocation density of different initial microstructures (tempered sorbite, lath martensite, bainite, and granular pearlite), this work reveals the microscopic mechanisms by which pretreatment inhibits grain growth. The findings provide critical insights for the industrial application of laser-induction hybrid phase transformation technology in heavy-duty bearings.

## 2. Materials and Methods

### 2.1. Experimental System

**Figure 1:**
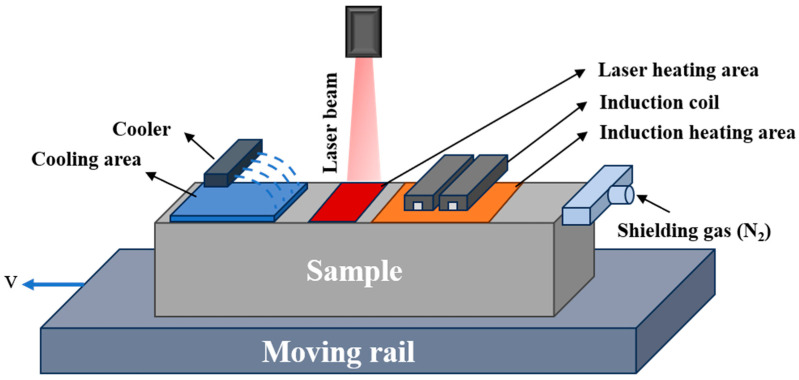
Schematic diagram of the laser-induction hybrid phase transformation process.

The experiments were conducted using a laser-induction hybrid system built by our labs, as shown in Figure 1, which mainly included a laser heating system, an electromagnetic induction heating system, a motion control system, a water-cooling device, a gas protection device, etc. The laser heating system used an LDF series continuous output fiber-coupled semiconductor laser (Laserline, Mülheim-Kärlich, Germany), which has a maximum laser output power of 6000 W, a laser wavelength range of 940~1090 nm, a spot shape for the rectangle, and an adjustable spot size range of 15~80 mm. The induction frequency of the induction heating equipment was adjustable from 3 to 24 kHz, the maximum output power was 80 kW, and the induction coil consisted of a copper tube and a magnetic conductor. The surface needed to be polished and descaled before the start of the experiment, and the surface of each specimen was cleaned with anhydrous ethanol solution with a volume fraction of 99.9% to reduce the influence of stains, burrs, and oxidized film on the surface during the experiment. This was followed by blackening to improve the laser absorption on the material surface. The gas provided by the gas protection device was nitrogen, whose main functions were as follows: (1) to prevent the specimen surface from contacting with air under the action of high temperature leading to decarburization; (2) to prevent the flow of coolant to the position of the heat source action during the heating process, which would have affected the experimental effect.

### 2.2. Material Preparation

**Figure 2:**
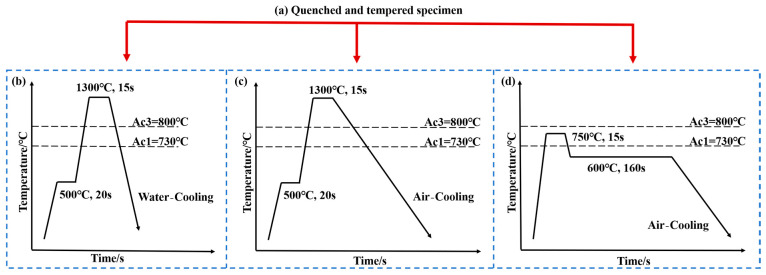
Schematic diagram of four laser-induction hybrid pretreatment processes: (**a**) quenched and tempered specimen; (**b**) laser-induction hybrid quenching (LIQ); (**c**) laser-induction hybrid normalizing (LIN); (**d**) laser-induction hybrid annealing (LIA).

The substrate material employed in this experiment was quenched and tempered 42CrMo steel (custom-produced to meet our requirements by the manufacturer), possessing a nominal chemical composition of 0.42C-0.2Si-0.65Mn-1.05Cr-0.2Mo-0.03Ni (wt.%). This condition was referred to as QT in this paper. The dimensions of the specimen were 55 mm × 50 mm × 110 mm. The critical temperatures of Ac1 and Ac3 were calculated to be 730 °C and 800 °C by JmatPro 6.0. In this work, parts of the microstructures were prepared using methods developed in our previous research [30], while the others were developed in this study. Three different microstructure specimens were prepared by adjusting the laser-induction hybrid heat treatment process. Figure 2 shows the flow of the different pretreatment processes.

The first pretreatment process was quenching and tempering (QT), where the specimen was quenched, followed by high-temperature tempering. This QT specimen was custom-processed to meet our requirements and supplied by the manufacturer.

The second pretreatment process was laser-induction hybrid quenching (LIQ), where the specimen underwent hybrid laser-induction heating with dual heat sources: first, it was rapidly heated by induction preheating; then, the preheated zone was laser-heated to above the austenitization temperature, immediately followed by water-cooling to room temperature, resulting in a surface layer of lath martensite.

The third pretreatment process was laser-induction hybrid normalizing (LIN), where the specimen was heated via hybrid laser-induction dual heat sources and then slowly air-cooled to room temperature. During this controlled cooling phase, the surface region remained within the bainite transformation temperature range (350–550 °C) for a prolonged duration due to the slow cooling rate, resulting in predominant bainite microstructure formation at the surface.

The final pretreatment process was laser-induction hybrid annealing (LIA): The specimen surface was heated to ~750 °C (slightly above A_c1_) using the hybrid heat source and held for 15 s. During cooling, a secondary induction scan was applied three times to precisely maintain the surface temperature around 600 °C (monitored in real time using a thermal imaging camera, FLIR, Wilsonville, America). This was followed by slow air-cooling to room temperature, promoting spheroidization and coarsening of carbides to yield a microstructure of granular pearlite. The laser-induction process parameters are shown in Table 1. Finally, the four pretreated specimens underwent laser-induction hybrid phase transformation under identical process parameters.

### 2.3. Microstructure Characterization and Austenite Grain Size Testing

At the end of the experiment, the surface of each specimen before and after laser-induction hybrid phase transformation was cut and sampled using electrical discharge machining wire cutting, and the depth-directed cross-section of the specimen was polished and later polished to give a mirror-like cross-section. All specimen sections were etched using a 4% volume fraction of nitric acid dissolved in alcohol. And the ZEISS EVO18 (ZEISS, Baden-Württemberg, Germany) scanning electron microscope (SEM) was used to analyze the tissue morphology characteristics before and after phase transformation. To analyze the crystal structure and orientation of the different pretreatment specimens, 10 mm × 10 mm × 3 mm samples were vibrationally polished, and a silica suspension was added during the polishing process to further eliminate the fine scratches and residual stresses on the surface remaining after mechanical polishing to obtain ultra-high-quality surfaces to meet the requirements of electron backscatter diffraction (EBSD) analysis. A Symmetry S3 EBSD detector was used for data acquisition, and AZtecCrystal software 2.1.2 was used for data processing and analysis.

To observe the austenite grain morphology after hybrid phase transformation, saturated picric acid solution was used to corrode the grain size of the specimens after laser-induction hybrid phase transformation, and the corrosion was carried out in a water bath environment at a water bath temperature of 65 °C for a corrosion time of 10 min, and the microstructure of the quenched layer and grain morphology were subsequently observed with an Axio Imager2 type Zeiss optical microscope for observation. The austenite grain size was counted using Image Pro Plus 6.0 software.

### 2.4. Hardness and Frictional Wear Testing

The HMV-2TADWXY automatic Vickers hardness tester (Shimadzu, Kyoto, Japan) was used to test the microhardness of the hardened layer at different depths of the cross-section, with an applied load of 2.942 N and a holding time of 15 s. Points were punched in the depth direction of the hardened layer cross-section, which was repeated three times, and the average value was taken. The depth of the hardened layer was evaluated by a hardness of 480 HV0.3 or more (a martensitic phase fraction of 50% or more was considered an effective hardened layer).

**Figure 3:**
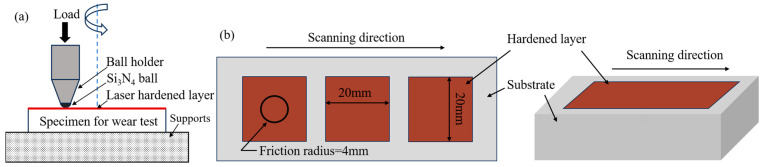
Schematic diagram of friction wear test: (**a**) schematic diagram of friction wear test; (**b**) dimensions and sampling location of friction wear specimen.

Friction wear experiments were conducted using an MMQ-02G-type friction wear testing machine (Yihua, Jinan, China) under dry friction conditions at room temperature, the principle of the testing machine is shown in Figure 3a. The quenched specimens for friction wear testing had dimensions of 20 mm × 20 mm × 10 mm, and the wear surface was progressively ground and polished until free of scratches, with the sampling location and specimen dimensions illustrated in Figure 3b. To simulate the actual working environment of heavy-duty bearings, the contact form adopts ball-disk point contact, and the friction pair consisted of Si_3_N_4_ ceramic balls with a diameter of 6.35 mm, which were fixed by the upper fixture and kept at rest under normal loading, with an applied load of 100 N, a rotational speed of 150 rad/min for the friction pair, a wear duration of 60 min, and a friction radius of 4 mm. Each test was repeated three times under the same conditions, and the friction data obtained were averaged over the three tests to ensure the stability and reliability of the results. The experimental design principles were derived from the Chinese standard GB/T 12444-2006 “Metallic Materials-Wear Test Method” [31,32]. To characterize wear mechanisms, the morphology of wear scars was analyzed using a ZEISS EVO18 (Zeiss, Baden-Württemberg, Germany) scanning electron microscope (SEM) and an energy dispersive spectrometer (EDS), focusing on features such as scratch depth, plastic deformation, and material transfer. The 3D cross-sectional profile of the worn specimen was characterized using a Keyence VK-X1000 3D (Keyence, Osaka, Japan) confocal height measuring instrument, and the wear morphology was observed by SEM.

## 3. Results and Discussion

### 3.1. Microstructure Characteristics of Different Pretreatment Processes

**Figure 4:**
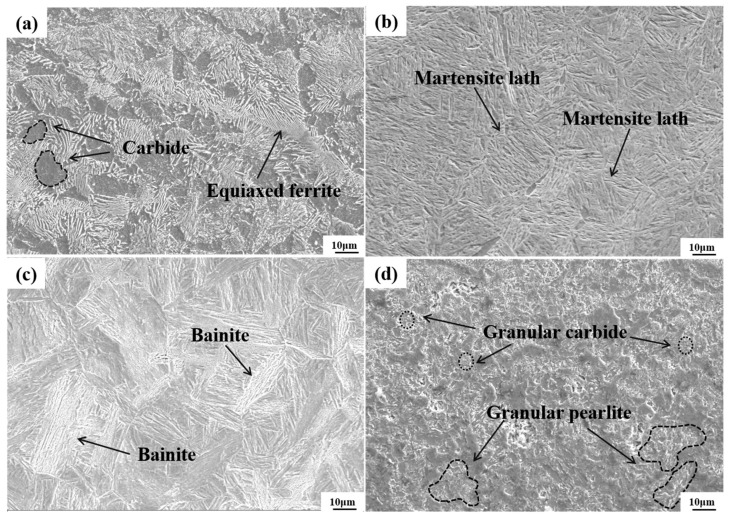
Microstructure after different pretreatment processes: (**a**) QT; (**b**) LIQ; (**c**) LIN; (**d**) LIA.

Figure 4 shows the microstructures of specimens prepared by four pretreatment processes before phase hardening. Significant differences in heating parameters, cooling paths, and phase transformation control strategies during thermal processing resulted in distinct microstructural characteristics among the four specimens.

The QT specimen was quenched and tempered at high temperatures. During this process, rapid quenching suppressed the precipitation of carbides, resulting in the formation of martensite. Subsequent tempering promoted the spheroidization and recrystallization of carbides, ultimately yielding a tempered sorbite microstructure composed of equiaxed ferrite and uniformly dispersed fine carbide particles. The LIQ specimen underwent coupled laser and induction dual-heat source interaction, rapidly heating its surface to 1200 °C near the melting point, followed by water quenching at a cooling rate surpassing the critical cooling rate of 42CrMo steel. This suppressed diffusional phase transformations, such as pearlite or bainite formation, ultimately producing lath martensite characterized by numerous parallel-aligned fine laths with uniform dimensions. The LIN specimen surface was heated to approximately 1200 °C via a hybrid heat source, followed by air-cooling at a rate of ~50 °C/s. This moderate cooling rate permits partial carbon diffusion. When the temperature enters the bainitic transformation range (350–550 °C), carbon partitioning between ferrite and austenite occurs, ultimately forming lower bainite. This microstructure is characterized by lenticular ferrite platelets with carbide particles oriented at specific angles between the platelets. The LIA specimen surface was heated to 750 °C, slightly above the Ac1 temperature, and held for 15 s, followed by air-cooling to 650 °C with isothermal holding at this temperature for 160 s. The prolonged annealing promoted cementite spheroidization and coarsening. Slow cooling allowed sufficient carbon diffusion and precipitation, ultimately forming a granular pearlite microstructure characterized by a ferrite matrix embedded with spheroidal carbide particles.

### 3.2. Hardened Layer Surface Microstructure

**Figure 5:**
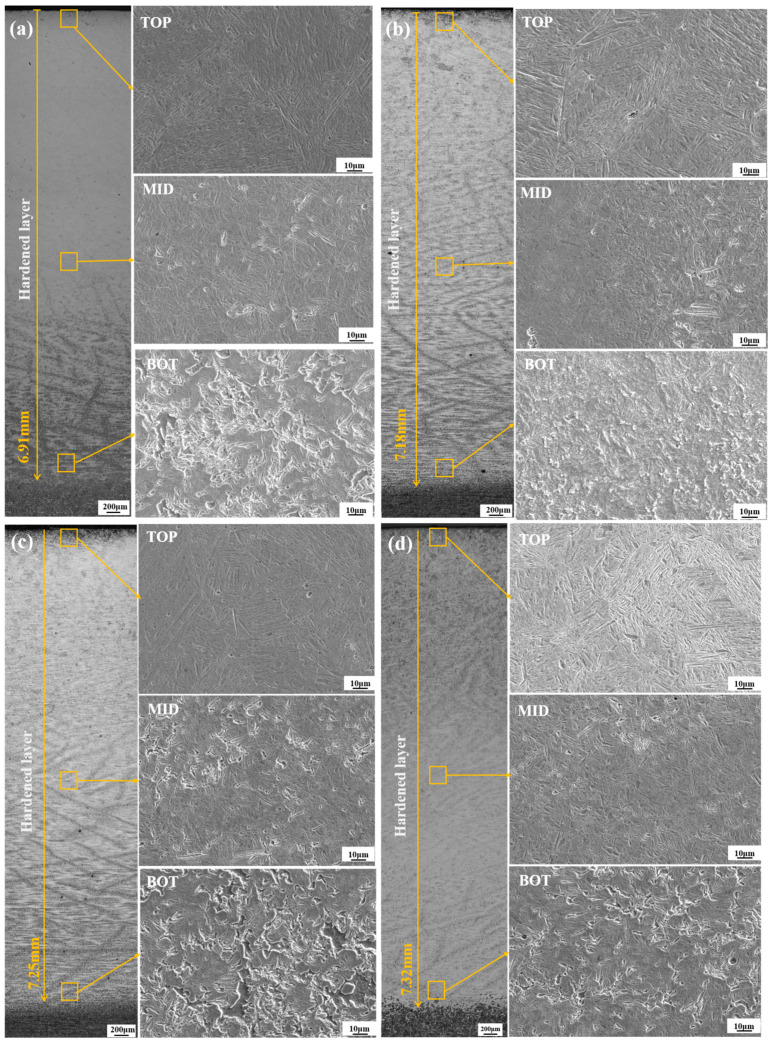
Microstructure and morphology of hardened layer at different positions after laser-induction hybrid phase transformation of specimens with different pretreatment processes: (**a**) QT; (**b**) LIQ; (**c**) LIN; (**d**) LIA.

To investigate the effects of the pretreatment processes on the microstructure of the hardened layer of 42CrMo steel, laser-induced hybrid phase transformation was performed on four differently pretreated specimens under identical conditions. The microstructures at different locations in the depth direction were then compared, and the results are shown in Figure 5. The variation in the temperature gradient across the specimen’s depth resulted in distinct microstructures at different depths. The microstructure at the top of the strengthened layer exhibited a more pronounced lath martensite organization in the hybrid-quenched specimens. This organization consisted of thin slat units arranged parallel to the austenite grains forming slat bundles. The separation between these slat bundles was marked by the presence of substantial angular grain boundaries, with the slat bundles themselves separated by smaller angles. A proto-austenite grain comprises multiple lath bundles, each containing distinct phases. As illustrated in the figure, quenched specimens with a post-pretreatment microstructure of bainite and granular pearlite (Figure 5c,d) formed shorter lath martensite bundles than those quenched specimens with a post-pretreatment microstructure of tempered sorbite and lath martensite (Figure 5a,b). The length of the lath martensite bundles was primarily influenced by the grain size of the original austenite. Consequently, the initial hypothesis that the grain size of austenite formed by austenitization would be smaller in the granular pearlite and bainite microstructures compared to the tempered sorbite and lath martensite microstructures produced under the same phase transformation conditions was formulated. In the middle region of the strengthened layer (3.5 mm from the surface), the SEM and the hardness curves demonstrate that the microstructures of the four quenched specimens in the middle region consist of a martensite phase and a small amount of tempered sorbite. The presence of the pearlite phase is attributed to insufficient laser energy delivery at greater depths, incomplete austenitization in certain regions, and variations in cooling rates during quenching. The lath martensite beams in this region are shorter than those at the top of the strengthened layer due to the lower quenching temperature in the middle of the strengthened layer compared to the top. This results in lower diffusion and migration rates of carbon atoms, leading to the formation of smaller austenite grains. Consequently, the lath martensite beams in this middle region (3.5 mm depth) are shorter than those at the top of the strengthened layer. In comparison with the bottom of the strengthened layer (6.5 mm from the surface), the content of martensite phases decreases, and the characteristics of the martensite morphology become more inconspicuous, and the lath martensite beams are shorter than those at the bottom of the strengthened layer (6.5 mm from the surface). This phenomenon is attributed to the lower phase transformation efficiency at greater depths due to insufficient laser energy input and reduced cooling rates. This is due to the lower phase transformation temperature at the bottom of the strengthened layer, which results in inadequate growth of the original austenite grains. Consequently, the content of the tempered sorbite phase increases further, accompanied by residual austenite and undissolved carbides. This outcome is attributed to the constrained energy delivery by the laser, a phenomenon that persists as the depth increases. Consequently, a substantial portion of the matrix tissue remains unfulfilled in terms of complete austenitization.

### 3.3. Austenite Grain Size and Analysis of Factors Affecting Austenite Growth

**Figure 6:**
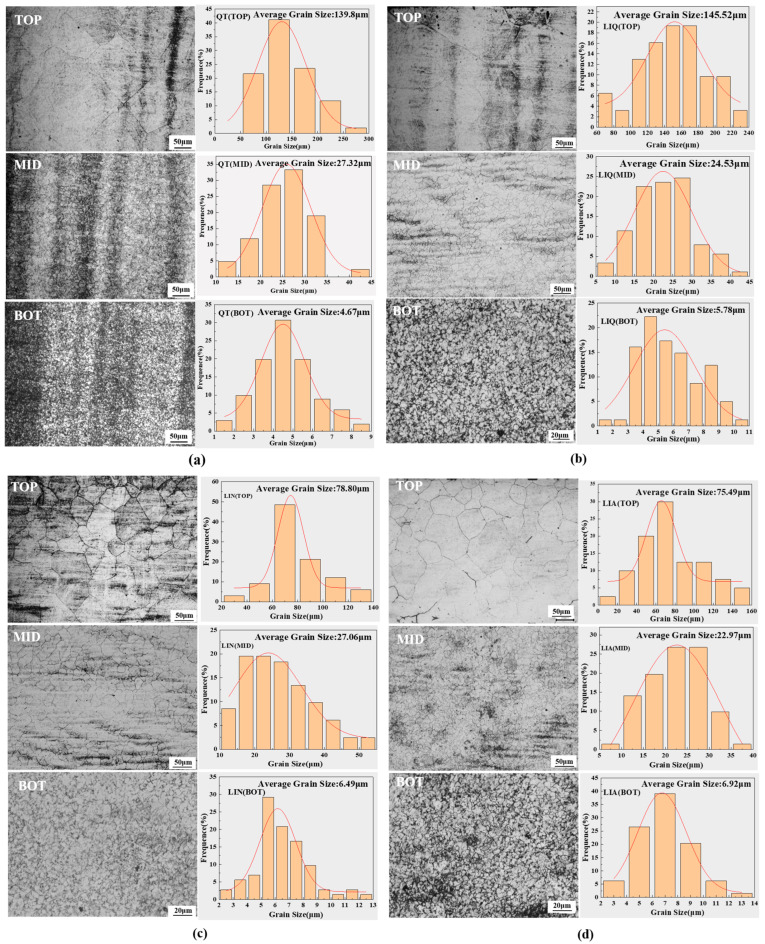
Grain morphology and grain statistics after hybrid phase transformation of specimens with different pretreatment processes: (**a**) QT; (**b**) LIQ; (**c**) LIN; (**d**) LIA.

The austenite grain size of 42CrMo after phase transformation with different pretreated specimens was measured using Image Pro Plus 6.0, and the results are shown in Figure 6. Figure 6a shows the average austenite grain size at the surface of the hardened layer in QT-pretreated specimens after laser-induction hybrid phase transformation. The average grain size was 139.80 μm, and the presence of mixed crystals was evident, attributable to segregation of the substrate composition, resulting in non-uniform alloy composition and, consequently, non-uniform grain size [33]. As illustrated in Figure 6b, the surface mean grain size measured approximately 145.52 μm for LIQ-pretreated specimens after laser-induction hybrid phase transformation. This was because secondary quenching eliminated the residual heterogeneous structure of the initial quenching, thereby promoting the uniform nucleation of austenite and effectively reducing the generation of the mixed crystal phenomenon. As shown in Figure 6c,d, LIN- and LIA-pretreated specimens after laser-induction hybrid phase transformation exhibited average surface grain sizes of 78.8 μm and 75.49 μm, respectively, which represented substantial decreases in the average grain size when compared with the previous two specimens.

Since austenite grain growth behavior is affected by different microstructures through the combined effects of carbide pinning, nucleation site density, alloying element distribution, and other factors [34], the next step was to characterize the different pretreated specimens in terms of crystal orientation, original ferrite grain size, angular grain boundary distribution, and dislocation density to analyze their influence on austenite grain nucleation and growth.

**Figure 7:**
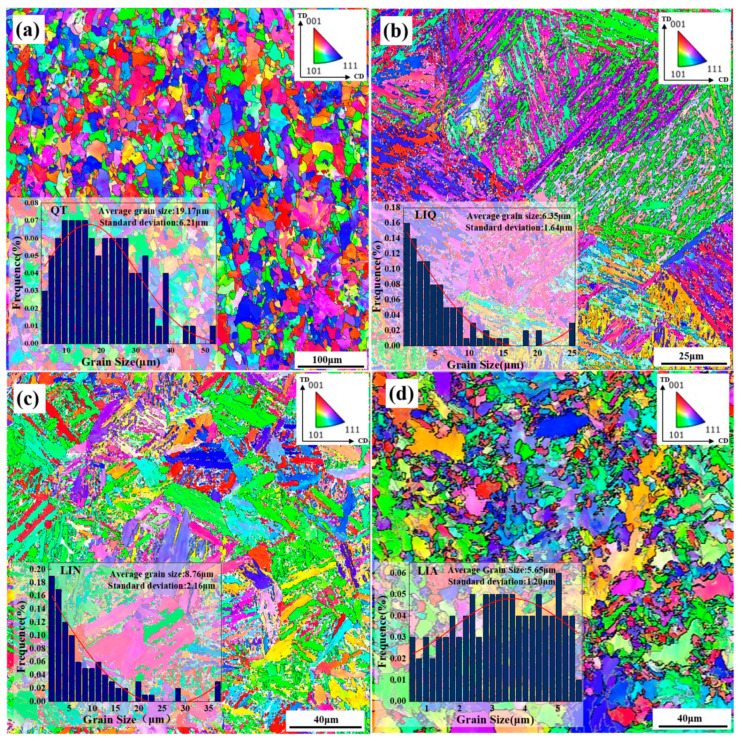
IPFs of different pretreated specimens and raw ferrite grain statistics: (**a**) QT; (**b**) LIQ; (**c**) LIN; (**d**) LIA.

**Figure 8:**
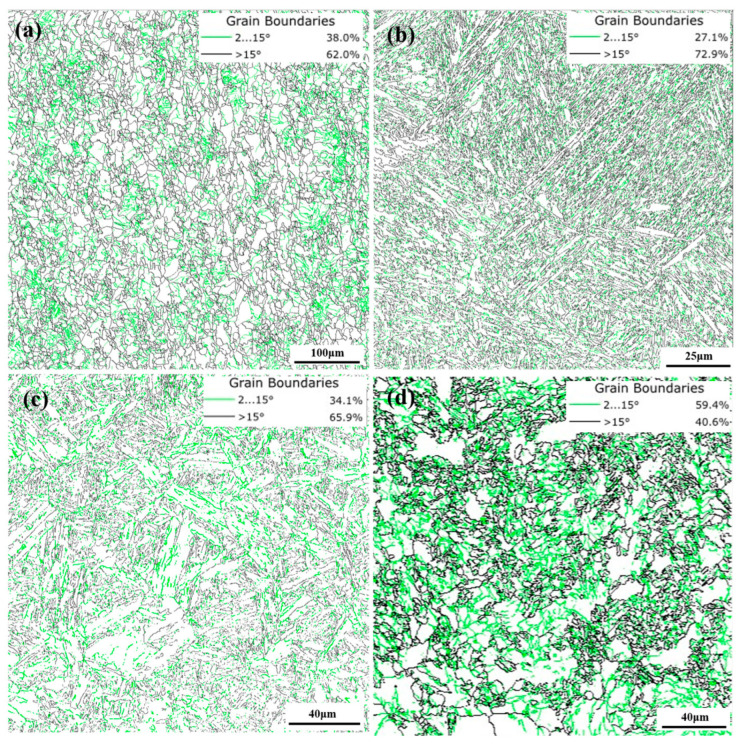
Distribution and proportion of low-angle and high-angle grain boundaries in different pretreated specimens: (**a**) QT; (**b**) LIQ; (**c**) LIN; (**d**) LIA.

Figure 7 presents the electron backscatter diffraction (EBSD) patterns of various pretreated specimens and their corresponding ferrite grain size distributions, featuring original average grain sizes of 19.17 μm, 6.35 μm, 8.76 μm, and 5.65 μm for the QT, LIQ, LIN, and LIA specimens, respectively. A comparative analysis shows the following: The QT specimen exhibits the largest initial grain size; the LIQ and LIN specimens show grain size reductions of 66.87% and 54.30%, respectively, compared to QT; and the LIA specimen demonstrates the finest grain structure with a 70.53% reduction relative to QT. Notably, all microstructures subjected to laser-induction hybrid heat treatment display refined ferrite grains, except for tempered sorbite. This grain refinement enhances austenite nucleation kinetics, as austenite preferentially nucleates at ferrite–carbide interfaces. Finer initial grains provide a greater interfacial area and a higher density of nucleation sites, thereby increasing the austenite nucleation rate. Critically, we observed that despite LIQ’s exceptionally fine original ferrite grain size (6.35 μm), its post-transformation hardened layer exhibited coarse austenite grains exceeding 140 μm (Figure 8d). This demonstrates that initial grain size alone is not the sole determinant of final austenite dimensions.

Figure 8 shows the distribution and percentages of low-angle and high-angle grain boundaries in different specimens, with the proportions of small-angle grain boundaries in QT, LIQ, LIN, and LIA specimens being 38.0%, 27.1%, 34.1%, and 59.4%, respectively. The results indicate that compared with the QT specimen, the proportion of small-angle grain boundaries in the LIQ, LIN, and LIA specimens changed to different degrees: those in the LIQ and LIN specimens decreased by 28.7% and 10.3%, respectively, while that in the LIA specimen increased by 36.1%. The influence of small- and large-angle grain boundaries on grain growth is mainly reflected in the following aspects: On the one hand, there are differences in grain boundary energy and migration ability. According to the Gibbs free energy minimization principle, the system tends to reduce the total interfacial energy, while small-angle grain boundaries have a correspondingly lower migration drive due to their lower initial energy state. During the hybrid phase transformation heating process, small-angle grain boundaries exhibit a low migration rate when the temperature reaches the austenitization temperature. This slow migration characteristic significantly limits the growth kinetics of austenite grains, making the organizational evolution process more gradual. On the other hand, the atomic arrangement at small-angle grain boundaries is relatively regular, with tighter interatomic bonding and narrower element diffusion channels, thus limiting element diffusion and consequently inhibiting the growth of austenite grains. In summary, LIA specimens with the highest proportion of small-angle grain boundaries are more favorable for suppressing austenite coarsening.

**Figure 9:**
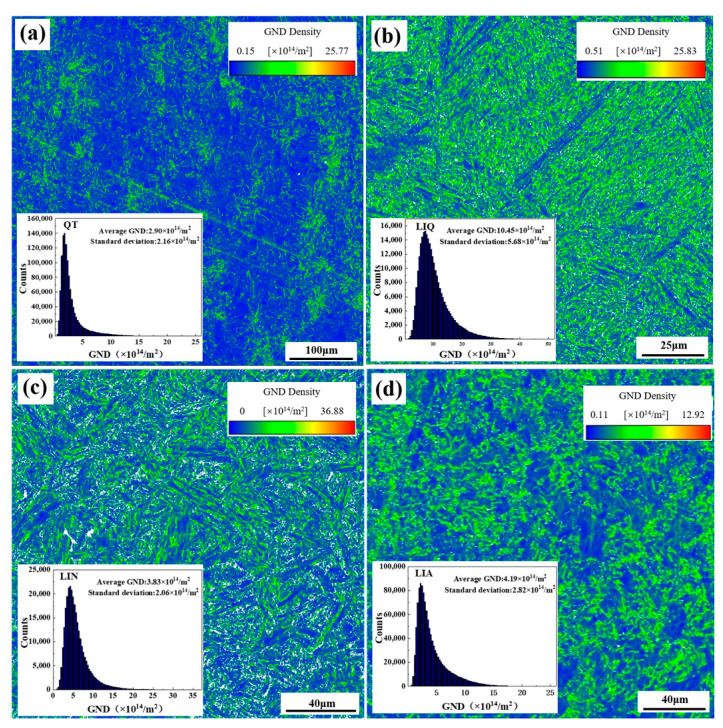
Statistics of GND distribution and average GND values of different pretreated specimens: (**a**) QT; (**b**) LIQ; (**c**) LIN; (**d**) LIA.

Figure 9 shows the geometrically necessary dislocation (GND) densities of different pretreated specimens. The results indicate that the GND densities of the QT, LIQ, LIN, and LIA specimens are 2.16 × 10^14^ m^−2^, 10.45 × 10^14^ m^−2^, 5.83 × 10^14^ m^−2^, and 4.19 × 10^14^ m^−2^, respectively. Compared with the QT specimen, the other three specimens show varying degrees of increase in GND density, with the LIQ specimen exhibiting the most significant increase (384.8%), indicating a substantially higher dislocation density in this microstructure. The analysis of GND densities reveals their significant influence on austenite grain size. From an energy perspective, a high dislocation density leads to considerable accumulation of lattice distortion energy. According to the Gibbs–Thomson effect, this stored energy provides an additional driving force for grain boundary migration during subsequent heat treatment. At the austenitization stage, these high-energy defect structures promote grain growth through three main mechanisms: (1) strain energy release through dislocation recombination lowers the activation energy for grain boundary migration; (2) disintegration of dislocation cell structures generates numerous mobile grain boundary segments; and (3) local stress gradients create chemical potential differences that accelerate atomic diffusion. In summary, while high dislocation density and lattice distortion energy provide a driving force for grain boundary migration and promote austenite grain growth, the LIQ specimen demonstrates that an excessive grain boundary driving force can lead to austenite grain coarsening despite the presence of fine initial ferrite grains.

In summary, finer initial grains provide a greater interfacial area and a higher density of nucleation sites, thereby increasing the austenite nucleation rate and potentially leading to finer austenite grains. However, the final austenite grain size is determined by a dynamic competition between nucleation and subsequent grain growth during austenitization. While a high nucleation density favors refinement, excessive driving forces for grain boundary migration, such as a high dislocation density (LIQ specimen) or a predominance of high-energy, high-angle grain boundaries, can promote rapid grain coarsening even with a fine initial microstructure. Conversely, microstructural features like a high fraction of low-angle grain boundaries effectively counteract the growth driving forces. Therefore, optimal pretreatment aims not only to refine the initial grains for increased nucleation sites but also to tailor the microstructural characteristics (dislocation density and grain boundary character distribution) to simultaneously suppress grain boundary mobility during the austenitization stage of the subsequent hybrid phase transformation. The results for the LIN and LIA specimens demonstrate the successful implementation of this strategy, achieving significant austenite grain refinement.

From the above results, it can be seen that the refined austenite grain size (75.5–78.8 μm) achieved by LIN and LIA is significantly better than that of the conventional quenched and tempered process, which is usually greater than 100 μm. Compared to other heat treatment processes, such as conventional furnace cycling heat treatment, finer grains can be obtained, but multiple thermal cycles are required, increasing complexity and cost. In contrast, our single-step LIN/LIA pretreatment achieves a certain degree of refinement (75.5–78.8 μm) which is much more industrially feasible.

### 3.4. Microhardness of Hardened Layer

**Figure 10:**
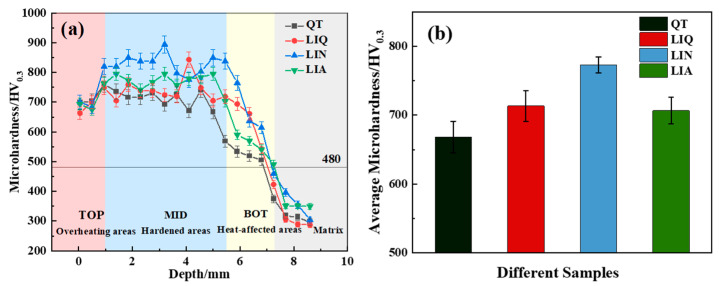
Distribution of microhardness values of the hardened layers of different hybrid quenched specimens: (**a**) microhardness change curve; (**b**) average microhardness.

The microhardness of specimens pretreated by QT, LIQ, LIN, and LIA, respectively, and subsequently subjected to laser-induction hybrid phase transformation was measured using a Vickers hardness tester (Shimadzu, Kyoto, Japan), with the results presented in Figure 10. The hardened layer depth for specimens pretreated with QT and then subjected to laser-induction hybrid phase transformation measured 6.91 mm. Similarly, specimens pretreated with LIQ, LIN, and LIA, followed by transformation, exhibited depths of 7.18 mm, 7.25 mm, and 7.32 mm, respectively. The surface microhardness values were 678.78 HV0.3 (QT-pretreated), 663.18 HV0.3 (LIQ-pretreated), 760.28 HV0.3 (LIN-pretreated), and 765.22 HV0.3 (LIA-pretreated). The average microhardness values were 612.04 HV0.3, 630.37 HV0.3, 691.09 HV0.3, and 667.59 HV0.3 for the QT-, LIQ-, LIN-, and LIA-pretreated specimens, respectively. The maximum depth variation between specimens was 0.41 mm. When combined with the austenite grain size data (Figure 6), these results indicate that specimens pretreated with LIN and LIA effectively prevent austenite grain coarsening at the hardened layer surface while maintaining sufficient hardening depth.

As demonstrated in Figure 10a, the hardened layer of the specimens post-laser-induction hybrid phase transformation could be broadly categorized into three zones: the surface superheated zone, the middle hardened zone, and the bottom heat-affected zone. The hardened layer’s change trend along the depth direction was always higher and then lower. This is because, to increase the depth of the hardened layer, the surface of the specimen is heated to a temperature much higher than the Ac3 line. Excessively high temperatures will lead to decarburization and oxidation of the surface of the specimen. The microhardness of the middle part of the hardened layer is higher than the surface of the hardened layer, on the one hand, due to the lower phase transformation temperature, which produces austenite grains of smaller size than the surface of the hardened layer, whereas, again, both are dominated by the lath martensitic phase. The smaller grain size improves the strength of the material according to the Hall–Petch formula. Conversely, the elevated martensite content within the middle of the hardened layer, as compared to the surface, facilitates the attainment of higher strength for the martensite phase within the middle.

The surface microhardness of LIN- and LIA-pretreated specimens after laser-induction hybrid phase transformation reached 760–765 HV0.3, significantly higher than the 663–679 HV0.3 range exhibited by QT- and LIQ-pretreated specimens subjected to identical transformation. This hardness enhancement is mainly attributed to fine-grain strengthening. The surface hardness obtained in this study on 42CrMo is in a similar high-level range to that of the 42CrMo laser-nitriding hybrid technique reported by Zhang [35], but a deeper hardened layer was achieved in this study.

### 3.5. Hardened Layer Friction and Wear Properties

**Figure 11:**
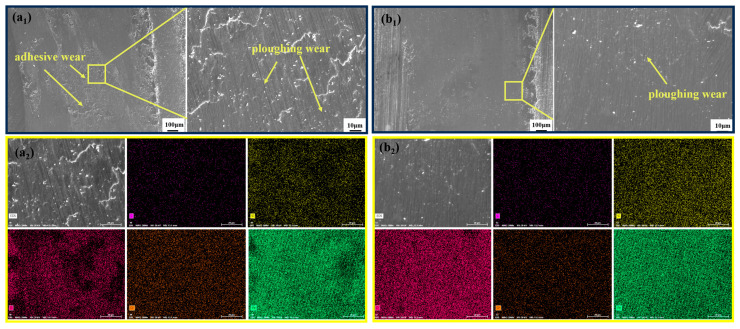
Scanning electron microscopy (SEM) and electron microanalysis (EDS) of friction wear furrows: (**a_1_**) SEM images for QT; (**a_2_**) EDS images for QT; (**b_1_**) SEM image of a laser-induction hybrid phase transformation pretreated for QT; (**b_2_**) EDS image of a laser-induction hybrid phase transformation pretreated for QT.

In Figure 11(a_1,_a_2_,b_1,_b_2_), the surface wear morphology of 42CrMo steel under only-QT and laser-induction hybrid phase transformation conditions is depicted in SEM images. The wear surfaces of both specimens were characterized by obvious ring-shaped scratches along the relative sliding direction. As shown in Figure 11(a_1_), the wear surface of the QT sample exhibits irregular material accumulation and tear-like pits. The EDS results of the QT sample reveal a significant presence of Si elements on the surface (Figure 11(a_2_)), which are attributed to the Si_3_N_4_ counterpart in the friction pair. This indicates material transfer and, combined with the morphological observations, confirms the occurrence of adhesive wear. In contrast, the hybrid phase transformation pretreated for QT (Figure 11(b_1_)) displays parallel grooves and ploughing marks aligned with the sliding direction. EDS analysis further detected foreign elements (Si and O), suggesting the involvement of abrasive particles. These findings collectively support the dominance of abrasive wear in the composite-quenched sample.

**Figure 12:**
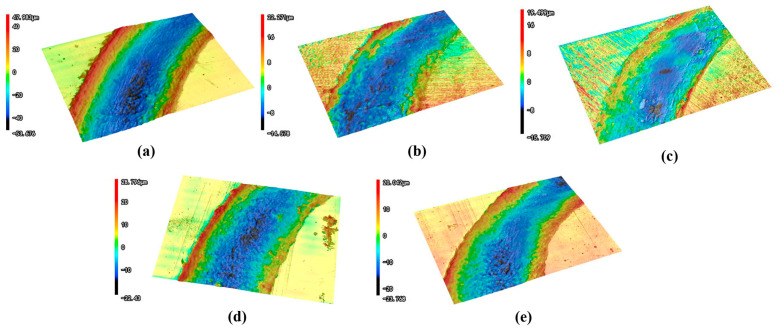
Wear morphology of hardened layer after laser-induction hybrid phase transformation of specimens with different pretreatment processes: (**a**) only QT; (**b**) QT; (**c**) LIQ; (**d**) LIN; (**e**) LIA.

**Figure 13:**
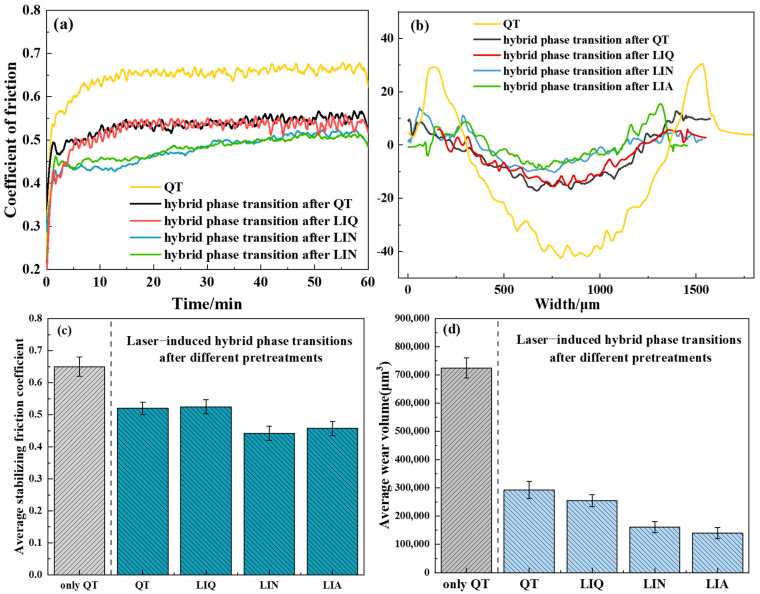
Friction and wear properties of specimens: (**a**) coefficient of friction; (**b**) plough groove morphology; (**c**) average stabilizing friction coefficient; (**d**) average wear volume.

As demonstrated in Figure 12, the wear profiles of the specimens exhibited a uniform and stable wear process, characterized by a smooth surface devoid of undulating furrow characteristics. Figure 13 presents data for quantitative analysis: the friction coefficient curves of representative specimens (Figure 13a), the wear profiles of representative specimens (Figure 13b), the average stabilized friction coefficients (Figure 13c), and the average wear volumes (Figure 13d). As demonstrated in Figure 13a, the friction coefficient curve fluctuated during the wear phase; however, it entered a stable wear phase when the wear time was greater than 5–10 min, at which point the coefficient of friction tended to stabilize. The presence of two distinct stages can be attributed to the initial wear, the friction vice, and the specimen surface micro-convex body acting as the primary contact. The role of point contact is evident, and the small contact area leads to stress, resulting in the friction vice and the specimen surface of the material crushing and undergoing plastic deformation. Consequently, the friction coefficient exhibits fluctuations. It was also found that the average stabilized friction coefficient and average volume area between different specimens exhibited significant variations, as shown in Figure 13c,d, and it was observed that the friction coefficient of the QT specimens without hybrid phase transformation stabilized at approximately 0.65 during the wear process, with a wear volume of 736,952.70 μm^3^. In contrast, specimens subjected to laser-induction hybrid phase transformation exhibited reduced surface friction coefficients and wear areas. The QT- and LIQ-pretreated specimens after hybrid transformation showed stabilized friction coefficients of 0.52 during wear testing, with wear volumes of 292,879.34 μm^3^ and 254,763.75 μm^3^, respectively, representing a 20% reduction in the friction coefficient compared to QT specimens. This reduction can be attributed to the surface of the specimen quenched by the laser-induction hybrid phase transformation containing a significant amount of martensite, and due to the martensite having a substantial solid-solution strengthening effect, the surface of the specimen underwent significant changes. Similarly, LIN- and LIA-pretreated specimens after laser-induction hybrid phase transformation demonstrated significantly enhanced wear resistance compared to the QT specimen. Furthermore, the coefficients of friction for both specimens were stabilized at approximately 0.45, which is approximately 30.7% lower than that of the QT specimen. The wear volumes for both specimens were 161,358.95 μm^3^ and 140,181.12 μm^3^, respectively. The difference in the wear resistance of the different specimens can be explained by the following mechanism.

**Figure 14:**
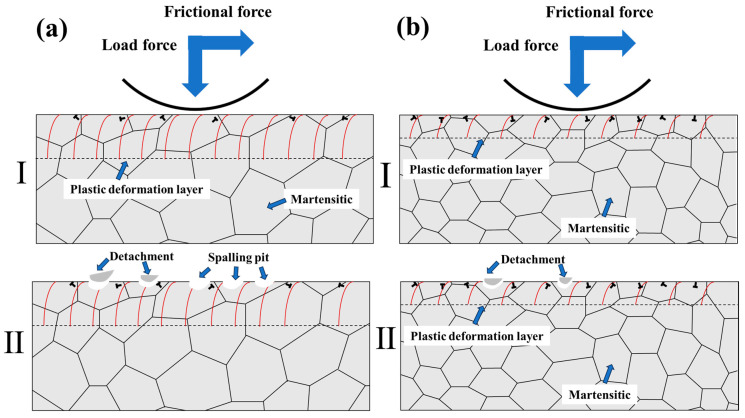
Schematic diagram of the wear mechanism of the hardened layer with different grain sizes: (**a**) coarse-grain structure; (**b**) fine-grain structure.

Figure 14 presents a schematic diagram of the surface wear mechanisms in hardened layers with different grain sizes. On the one hand, according to the material strengthening mechanism described by the Hall–Petch relationship, grain refinement significantly increases the number of grain boundaries. These boundaries act as effective barriers to dislocation motion, substantially enhancing the material’s resistance to dislocation slip and consequently improving its overall hardness and yield strength. This theoretical prediction was confirmed by microhardness test results: the surface hardness values of LIN- and LIA-pretreated specimens after laser-induction hybrid phase transformation were significantly higher than those of the QT- and LIQ-pretreated specimens subjected to the same transformation process. The enhanced material strength effectively suppresses surface plastic deformation and micro-cutting phenomena, thereby reducing frictional energy dissipation and material loss. These observations demonstrate that finer grain sizes indeed produce stronger strengthening effects. On the other hand, during the friction process, the dense grain boundaries in fine-grained materials create physical barriers to dislocation slip. As understood from the grain boundary pinning effect, dislocations migrating toward grain boundaries require additional energy to either change their slip direction or activate new dislocation sources, thereby increasing resistance to plastic deformation. In coarse-grained materials, dislocations can slip over long distances to form deep grooves, whereas in fine-grained materials, dislocations are confined within individual grains, resulting in only superficial microplastic deformation. Furthermore, the fine-grained structure exhibits a higher work-hardening rate during wear due to interactions between dislocation pile-ups and grain boundaries.

The friction coefficient of LIN- and LIA-pretreated specimens after laser-induction hybrid phase transformation (0.45) represents a 30.7% reduction compared to QT specimens, surpassing the 25% reduction reported for laser-quenched 42CrMo steel [36]. This enhancement aligns with the findings of Huang et al. [26], who observed a 40% toughness improvement in normalized 28MnB5 steel due to martensite lath refinement. Notably, our LIN process achieves similar grain refinement effects without additional alloying, demonstrating the efficiency of microstructure design through hybrid pretreatment.

## 4. Conclusions

1. The four pretreatment methods (QT, LIQ, LIN, and LIA) resulted in distinct initial microstructures that strongly influenced austenite grain growth during laser-induction hybrid phase transformation. Tempered sorbite (QT) exhibited coarse austenite grains (139.8 μm) due to its large original ferrite grains and sparse nucleation sites. Lath martensite (LIQ) showed similarly coarse grains (145.52 μm) despite fine initial grains, as a high dislocation density and large-angle grain boundaries promoted rapid grain boundary migration during austenitization. Bainite (LIN) and granular pearlite (LIA) achieved refined grains through dense nucleation sites and Zener pinning by undissolved carbides; the average grain sizes of the hardened layer were 75.49 μm and 78.8 μm, respectively. Granular pearlite further benefited from a high proportion of small-angle grain boundaries (59.4%), significantly hindering grain growth.
2. All specimens achieved hardened layer depths exceeding 6.9 mm. Among these, specimens pretreated with LIN and LIA prior to transformation exhibited the highest surface microhardness values (760.3 HV0.3 and 765.2 HV0.3, respectively), representing a 12–15% increase over specimens pretreated with QT and LIQ. This improvement was mainly due to finer martensitic structures formed from refined austenite grains, validating the effectiveness of pretreatment in enhancing surface hardness via grain refinement.
3. Specimens pretreated with LIN and LIA prior to laser-induction hybrid phase transformation demonstrated shallow-scratch abrasive wear characteristics, achieving friction coefficients of 0.45 with wear volumes of 161,358.95 μm^3^ and 140,181.12 μm^3^, respectively. This represents reductions of 30.7% in friction coefficient and 80% in wear volume compared to only-QT specimens. Conversely, specimens pretreated with QT and LIQ followed by transformation exhibited higher friction coefficients (0.52) and wear volumes exceeding 2.7 × 10^5^ μm^3^, confirming the direct correlation between grain refinement and wear resistance. Grain refinement enhances the grain boundary density, which effectively impedes dislocation motion and improves the material’s resistance to plastic deformation.

In summary, the LIN and LIA pretreatments proposed in this study provide an effective way to solve the grain coarsening problem in laser-induction hybrid deep phase change hardening which is relatively simple and efficient compared to other grain refinement methods and achieves high hardness and significantly better wear resistance than conventional pretreatments (QT) while obtaining an ultra-deep hardened layer (greater than 6.9 mm) with advanced surface hardening techniques, showing good engineering application prospects.

## Figures and Tables

**Table 1:** Laser-induction hybrid pretreatment process parameters.

Pretreatment	Laser Power (kW)	Spot Size	Scanning Speed (mm·s^−1^)	Induction Power (kW)	Cooling Method	Target Microstructure
LIQ	5	55 mm × 15 mm	2.5	40	Water	Lath martensite
LIN	5	55 mm × 15 mm	2.5	40	Air	Bainite
LIA	2	55 mm × 15 mm	1.5	28-20-20	Air	Granular pearlite

## Data Availability

The original contributions presented in this study are included in the article. Further inquiries can be directed to the corresponding author.

## Abbreviations

The following abbreviations are used in this manuscript:

QT	Quenched and Tempered
LIQ	Laser-Induction Hybrid Quenching
LIN	Laser-Induction Hybrid Normalizing
LIA	Laser-Induction Hybrid Annealing
